# Bacterial cellulose as a potential bioleather substitute for the footwear industry

**DOI:** 10.1111/1751-7915.13306

**Published:** 2018-08-22

**Authors:** Concha García, María Auxiliadora Prieto

**Affiliations:** ^1^ Patent Shoes SL Calle Feijóo, 18 28010 Madrid Spain; ^2^ Polymer Biotechnology Lab Biological Research Center Spanish National Research Council (CIB‐CSIC) C/Ramiro de Maeztu, 9 28040 Madrid Spain

## Abstract

Shoe patterns and a sole made of BC, baby shoe made of BC and, on the right BC material dyed in red. (prototype made by www.patent-shoes.com).

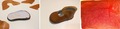

All consumer goods – including fashion products – use up resources; there is no exception to this rule. According to Mark Sumner of the University of Leeds (UK), *the world of fashion has some major sustainability problems. By 2030, it is predicted that the industry's water consumption will grow by 50 per cent to 118 billion cubic metres, its carbon footprint will increase to 2,791 tonnes, and the amount of waste it creates will hit 148 tonnes* (https://www.independent.co.uk/life-style/fashion/it-may-not-be-possible-to-slow-down-fast-fashion-so-can-the-industry-ever-be-sustainable-a7970031.html). These predictions appeared despite significant progress being made by brands and retailers to minimize their impact. Many are following sustainable initiatives to reduce their use of energy and chemicals throughout the supply chain. Attempts are also being made to reduce water consumption, e.g., through the use of new dyeing technologies (http://globalfashionagenda.com/wp-content/uploads/2017/05/Pulse-of-the-Fashion-Industry_2017.pdf).

As the manufacture of leather is dependent on animal skins, the global leather goods business is no stranger to the issue of sustainability. Raising and slaughtering the millions of animals whose skins feed the industry are inefficient, are cruel, and come with a huge environmental cost. A single pair of leather boots requires the use of 50.2 m^2^ of land and 25,000 L of water, although if the wastewater from the leather tanning process passes through a treatment plant, and this demand can be reduced to 14,500 L (https://www.foe.co.uk/sites/default/files/downloads/mind-your-step-report-76803.pdf).

## Economic importance of the leather industry

From raw hides to finished garments, the global leather trade was worth US$77.5 billion in 2010; for 2018, its predicted worth is US$91.2 billion (the 2013–2018 annual growth rate being 3.4%). In Europe, the leather and related goods sector comprises about 36 000 enterprises, which together have a turnover of €48 billion and employ around 435 000 people. The footwear industry, which accounts for 41% of this, is the largest market segment (http://ec.europa.eu/growth/sectors/fashion/leather/eu-industry/).

The increased focus on animal rights plus the stringency of laws governing the manufacture of real leather are, however, propelling demand for synthetic substitutes. The global syntheztic leather market was worth US$22.13 billion in 2015; its predicted worth for 2021 is US$33.54 billion, and for 2025 some US$85.05 billion (2016–2021 compound annual growth rate: 7.20%) (https://www.businesswire.com/news/home/20170620005839/en/Synthetic-Leather-Market-Reach-85-Billion-2025). Growing demand from major end‐use industries such as the footwear, furnishing and automotive industries is expected to drive the market. However, the harmful environmental effect of polyurethane (PU) and polyvinyl chloride (PVC) processing is a major problem (https://www.researchandmarkets.com/reports/3985073/synthetic-leather-artificial-leather-market-by#relb0). New leather substitutes are therefore needed.

One start‐up, Modern Meadow, is developing a ‘leather growing’ technique using a strain of yeast genetically engineered to produce bovine collagen. Vegan leather enterprises, which produce artificial leather from vegetable sources such as apples (The Apple Girl), pineapples (Ananas Anam), grapes (VEGEA), mushrooms (Grado Zero Espace), soy (XXLab), paper (Paper No. 9), corn (Coronet), cork (Pelcor) and tea (Iowa University and ScobyTec), are also springing up. Cellulose of plant origin has long been used to make textiles (rayon, e.g., is made from pulp or cellulose), and scientists have developed highly processed forms of this material (Dufresne, 2017) that provide strong, light and durable textiles with which to make clothing. Many large companies – among them Land Rover, Tesla and H&M – are taking advantage of these new sourcing opportunities (https://vegnews.com/2017/8/land-rover-exec-condemns-leather-car-interiors). Major sportswear brands have also shown interest in vegan alternatives to oil‐based materials, a consequence of the growing ecological awareness of the need to avoid materials such PVC. For example, Puma, Adidas and Nike are developing an eco‐leather made from natural fibres mixed with plant oils developed at the University of Delaware (https://inhabitat.com/ecouterre/renewable-eco-leather-made-from-plant-oils-is-a-fashion-game-changer/; http://www.udel.edu/udaily/2014/dec/wool-epa-121113.html). Moreover, Adidas has recently developed prototype eco‐trainers made almost entirely from waste recovered from the sea, and plans to begin using recycled fibres (https://www.independent.co.uk/life-style/fashion/adidas-ultraboost-x-parley-ocean-pollution-trainers-plastic-a7701486.html).

## Bacterial cellulose as a potential leather substitute

Some bacteria naturally produce cellulose [bacterial cellulose (BC)]. If produced biotechnologically in large quantities, it might afford an alternative to plant cellulose. BC is a remarkable material; it is malleable, biocompatible, over 10 times stronger than plant cellulose, and it is highly hydrophilic (it can store over 90% its own weight of water) (Sun *et al*., 2010). BC is already used commercially in high‐end acoustic products, in medical wound dressings, and to make many other goods (Lee *et al*., 2014). At the laboratory scale, it has even been used to create artificial blood vessels and biodegradable tissue scaffolds, and has shown promise in organic light‐emitting diode displays, flexible electrodes, sensors and other devices (Hu *et al*., 2014). Goodyear Tire, Kimberly‐Clark Corp. and Sony Corp., among others, have all filed patents involving the use of BC in some way.

The idea of BC as a potential leather substitute rests on the industrial production of cellulose fibres by members of the genera *Komagataeibacter* (bacteria ingested as part of kombucha tea and other fermentations, and which enjoy *Generally Regarded As Safe* [GRAS] status), e.g., by the model strain *K. xylinum* (Kubiak *et al*., 2014). These microorganisms, either alone or in combination with other bacteria and yeasts, aerobically produce pellicles of cellulose that accumulate in the extracellular medium. These can be generated at desired thicknesses and when dried produce a resilient leather‐like material with properties that resemble the type of animal leathers used in the footwear industry. Large quantities of these pellicles could be obtained via the industrial scale fermentation of simple nutrients. BC has the potential to be functionalized with therapeutic compounds, antimicrobial agents and even cosmetic products for foot care. However, despite the interest shown in BC and other polymers by industry, academia (Lee *et al*., 2014) and the art and fashion worlds (Rognoli *et al*., 2015), they have never been optimized for use by the footwear industry.

The tactile properties of BC are similar to those of fine finished leather; i.e., it is very soft and stretchable. These properties are important for the user's well‐being and comfort, but for people with diabetes, it is crucial: even minor cuts and scrapes need to be avoided. As pointed in the Strategic Research Agenda for the foot care sector 2015–2020, there are around 366 million diabetics and this is expected to rise to 552 million by 2030 (9.9% of the adult population worldwide). Some 15% of diabetics suffer foot ulcers, and 84% of amputations of the lower limbs in diabetics are related to their development of foot ulcers. Patients with diabetic foot syndrome therefore need footwear that reduces the risk of skin damage. In addition, by 2025 more than 20% of Europeans will be 65 years old or over, and there will be a particularly large increase in the number of over 80‐year‐olds. These groups also need to avoid foot infections, and thus, research into the development of new materials aimed at reducing the risk of foot infections for these groups has been identified as a major challenge by different forums (http://www.sohealthyproject.eu/results/main-results-library/publications/50-strategic-research-agenda).

The functionalization and modification of BC have been achieved through chemical or mechanical alteration of the polymer, and by making adjustments to the conditions of cultivation (Hu *et al*., 2014; Lee *et al*., 2014). By controlling the growth of the producing bacteria, the BC generated could be tailored to have properties desired by the footwear industry. Sheets of BC measuring 40 × 40 cm – a size compatible with footwear manufacturing requirements – could also be produced. If the solubility limitations of BC can be overcome, a BC‐based printable fluid might be produced and used to 3D print shoes (the Feetz company is already printing shoes using foam as the base material, https://feetz.com/3d-printing).

## Benefits of the implementation of BC in the footwear industry

Both the production and disposal of footwear are environmentally unfriendly. Certainly, stock raising is a significant source of greenhouse gases, and it consumes fossil fuels and water supplies. Further, footwear manufacturing normally involves harmful materials such as chromium‐tanned leather, chemical‐based adhesives and synthetic rubbers. Leather shoes themselves can even be toxic due to the presence of chromium VI compounds formed through the oxidation of chromium III used in the tanning process. In contrast, BC production should be much more bio‐economically sustainable – especially as the limited amount of land suitable for stock raising means the supply of leather will eventually meet a ceiling, resulting in higher leather prices (United Nations Industrial Development Organization, 2009). Further, the use of plant‐derived water‐soluble dyes should render BC‐based footwear hypoallergenic. Finally, skin disposal management is expensive, and the accumulation of waste in landfills has a hugely negative environmental impact. BC, in contrast, can be expected to undergo rapid and eco‐friendly biodegradation, with no leaching of toxic compounds to groundwater. Even a small market penetration of BC as a leather substitute could therefore result in a reduced demand for animal hides, fewer greenhouse gas emissions and diminished tanning‐associated toxicity.

Bacterial cellulose could also act as a leather substitute in the clothing, furniture and automotive industries. Indeed, factory‐grown leather promises several advantages over animal skins. For example, unlike real skins, BC sheets could be produced with straight edges, be devoid of scars, marks and other defects, and would not vary from batch to batch in the way that natural skins vary between animals. All these facets of BC reduce waste and improve quality.

## Challenges and outlook

Bacterial cellulose is currently very expensive, which renders it an unrealistic alternative to leather for now. The main factor contributing to its high production cost is the synthetic media required to culture the bacteria. However, strategies are being designed for the effective and economic production of BC using food and industrial wastes as sources of nutrients (Urbina *et al*., 2017). It is also critical that the reactor conditions of temperature, pH, dissolved oxygen content, medium composition and stirring speed, etc., be optimized if production costs are to fall. The design of the reactor is key (reviewed in Islam *et al*., 2017) as the best quality BC is generated in static cultures without agitation and in which oxygen transfer is limited. The development of genetically modified strains might improve the structural characteristics of BC and its yield. Synthetic biology strategies are now being used to optimize BC‐producing strains (Florea *et al*., 2016).

The inherent wettability and liquid‐absorbing capacity of BC are beneficial in some applications, but are crucial drawback in shoe manufacture; it is hard to imagine boots to which water sticks rather than rolling off. However, some living organisms possess superhydrophobic surfaces that are being imitated by polymer chemists, and these may be bonded to BC to generate materials with super‐antiwetting and even self‐cleaning properties (Liu *et al*., 2016). The surface chemical modification of cellulose nanomaterials to promote their compatibility with low polarity media is being widely investigated. Covalent functionalization, which generally involves reactive hydroxyl groups on the BC surface, is a favoured strategy. Melt processing, such as extrusion or injection moulding, may also be viable at the industrial level. However, the inherent incompatibility between hydrophilic cellulose and generally hydrophobic polymer matrices, as well as thermal stability issues, needs to be addressed (Dufresne, 2017).

Fast fashion is seen by many as the fundamental cause of all the sustainability issues the fashion industry faces. Consumption, and in particular fashion consumption, is quite irrational. Purchase decisions are more likely to be driven by desires linked to pleasure and excitement than by ethical issues. The first challenge in making fashion more sustainable might not therefore be to find ways of controlling our irrational behaviours, but to find an ethical means to embrace them. In this context, the production of bio‐based materials such as BC is not unattractive. If it can be made cheaply enough, it might provide the footwear industry with an ethical alternative to leather – one that might even allow us to embrace our fickle fashion behaviour.

## Conflict of interest

None declared.
